# Supplementary material for: Comparative multiomics analysis of cell physiological state after culture in a basket bioreactor
Source: Sci Rep. 2022 Nov 23;12:20161. doi: 10.1038/s41598-022-24687-4 (PMC9686226; doi:10.1038/s41598-022-24687-4)

969297:+@NW_023666041.1:152972650:152972814:+@NW_023666041.1:152976192:152976347:+@NW_0236[illegible]

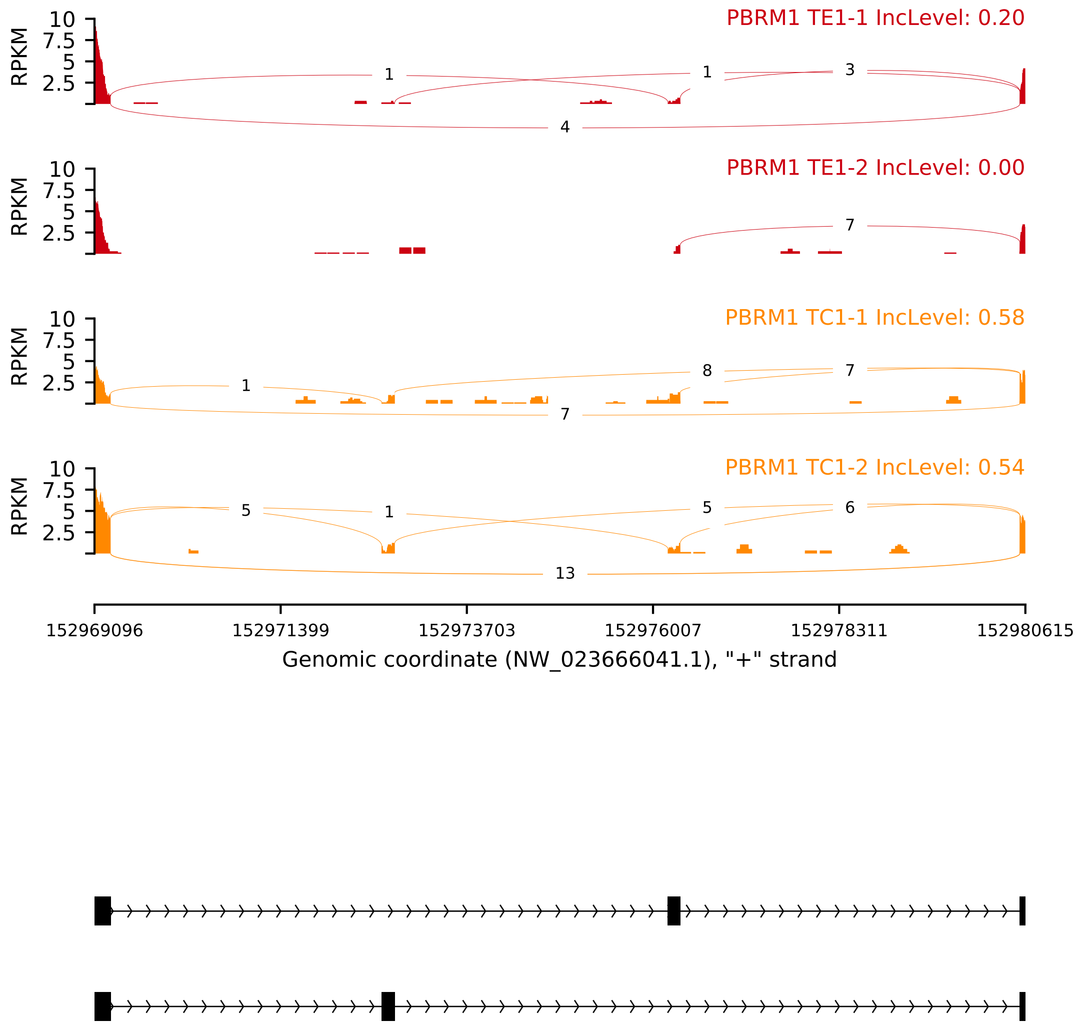

Supplement: Supplementary file 1 — Supplementary Information 1. [file 41598_2022_24687_MOESM1_ESM.zip › raw data/Transcriptomic raw data/7.AS/2.dasplot/TE1vsTC1/MXE/20_PBRM1_NW_023666041.1_152969096_152969297_+@NW_023666041.1_152972650_152972814_+@NW_023666041.1_152976192_152976347_+@NW_023666041.1_152980546_152980616_+.pdf]
